# Tumor penetrating peptides inhibiting MYC as a potent targeted therapeutic strategy for triple-negative breast cancers

**DOI:** 10.1038/s41388-018-0421-y

**Published:** 2018-08-03

**Authors:** Edina Wang, Anabel Sorolla, Paula T. Cunningham, Heique M. Bogdawa, Samuel Beck, Emily Golden, Robert E. Dewhurst, Laura Florez, Mark N. Cruickshank, Katrin Hoffmann, Richard M. Hopkins, Jonghwan Kim, Andrew J. Woo, Paul M. Watt, Pilar Blancafort

**Affiliations:** 1 Harry Perkins Institute of Medical Research, QEII Medical Centre, Nedlands and Centre for Medical Research, The University of Western Australia, Crawley, WA 6009 Australia; 2 School of Human Sciences, The University of Western Australia, Crawley, WA 6009 Australia; 3 Phylogica Pty Ltd, Subiaco, WA 6008 Australia; 4 Telethon Kids Institute, The University of Western Australia, Subiaco, WA 6008 Australia; 5 Department of Molecular Biosciences, The University of Texas at Austin, Austin, TX 78712 USA; 6 MDI Biological Laboratory, Kathryn W. Davis Center for Regenerative Biology and Medicine, Salisbury Cove, ME 04672 USA

**Keywords:** Breast cancer, Drug delivery, Targeted therapies

## Abstract

Overexpression of *MYC* oncogene is highly prevalent in many malignancies such as aggressive triple-negative breast cancers (TNBCs) and it is associated with very poor outcome. Despite decades of research, attempts to effectively inhibit MYC, particularly with small molecules, still remain challenging due to the featureless nature of its protein structure. Herein, we describe the engineering of the dominant-negative MYC peptide (OmoMYC) linked to a functional penetrating ‘Phylomer’ peptide (FPPa) as a therapeutic strategy to inhibit MYC in TNBC. We found FPPa-OmoMYC to be a potent inducer of apoptosis (with IC_50_ from 1–2 µM) in TNBC cells with negligible effects in non-tumorigenic cells. Transcriptome analysis of FPPa-OmoMYC-treated cells indicated that the fusion protein inhibited MYC-dependent networks, inducing dynamic changes in transcriptional, metabolic, and apoptotic processes. We demonstrated the efficacy of FPPa-OmoMYC in inhibiting breast cancer growth when injected orthotopically in TNBC allografts. Lastly, we identified strong pharmacological synergisms between FPPa-OmoMYC and chemotherapeutic agents. This study highlights a novel therapeutic approach to target highly aggressive and chemoresistant MYC-activated cancers.

## Introduction

The MYC transcription factor, regulating 15% of all annotated genes [1], is recognized to play essential cellular roles in all cells by promoting cell proliferation [2], growth [2], adhesion [3], metabolism [4], angiogenesis [5], differentiation [3], apoptosis [6], and metastatic dormancy [7]. Deregulation of oncogenic *MYC* expression is observed in greater than 70% of human malignancies [7] and occurs by several mechanisms, notably gene amplification and gene overexpression.

Importantly, *MYC* is amplified in 53% of basal-like breast cancers, which are triple-negative breast cancer (TNBC) [8] lacking the expression of estrogen receptor, progesterone receptor and HER2 [9]. Consequently, TNBC patients show elevated levels of MYC expression, which correlates with tumor progression with poor prognosis [4, 10]. It has been shown that MYC is overexpressed preferentially in TNBCs of the basal like subtype due to mechanisms such as copy number amplification (in ~53% of all basal-like breast cancers), changes in MYC promoter transcriptional regulation and protein stability [5, 11]. It has been suggested that MYC drives specific pathways in different breast tumors. While in ER− disease MYC overexpression may drive glucose metabolism to satisfy the proliferative demand of these tumors [12, 13], in ER+ disease MYC is associated with enhanced translation machinery and anti-oestrogen resistance [12, 14].

Despite its central oncogenic role, with the absence of a well-defined ligand-binding pocket, MYC has traditionally been considered a difficult-to-drug target [15]. Moreover, selective small inhibitors disrupting the protein–protein interactions involved in the MYC signaling network have been developed. BET bromodomains inhibitors, such as JQ1 [16, 17] and OTX015 [18], competitively bind to the acetyl-lysine recognition pocket of BET bromodomains reducing the recruitment of transcriptional activators [17]. Notably, BET inhibitors (BETis) downregulate MYC transcriptional activity [17] in TNBCs [19], sarcomas [16], and leukaemias [18]. However, these inhibitors affect hundreds of targets resulting in poor selectivity and quick tumor-adaptative response in acute myeloid leukemia models and other cancer models, causing MYC levels to remain unchanged [19–21].

More selective strategies to inhibit MYC have focused on small molecule inhibitors disrupting the interaction between MYC and its direct binding partner, the transcription factor MAX [22]. The IIA6B17 [23], 10058F4 [24], 10074-G5 [24] compounds were derived from a peptidomimetic library and have been shown to specifically block MYC-MAX dimerization both in vitro and in vivo. A new MYC inhibitor, KJ-Pyr-9 [25], was similarly isolated from a Kröhnke pyridine library screening and demonstrated growth inhibition of *MYC*-amplified TNBC xenografts [25]. However, inhibiting MYC-MAX interaction in vivo has been limited by fast metabolism, poor potency, resistance mechanisms, and poor tumor penetrability of these small molecule inhibitors [26–30].

**Fig. 1:**
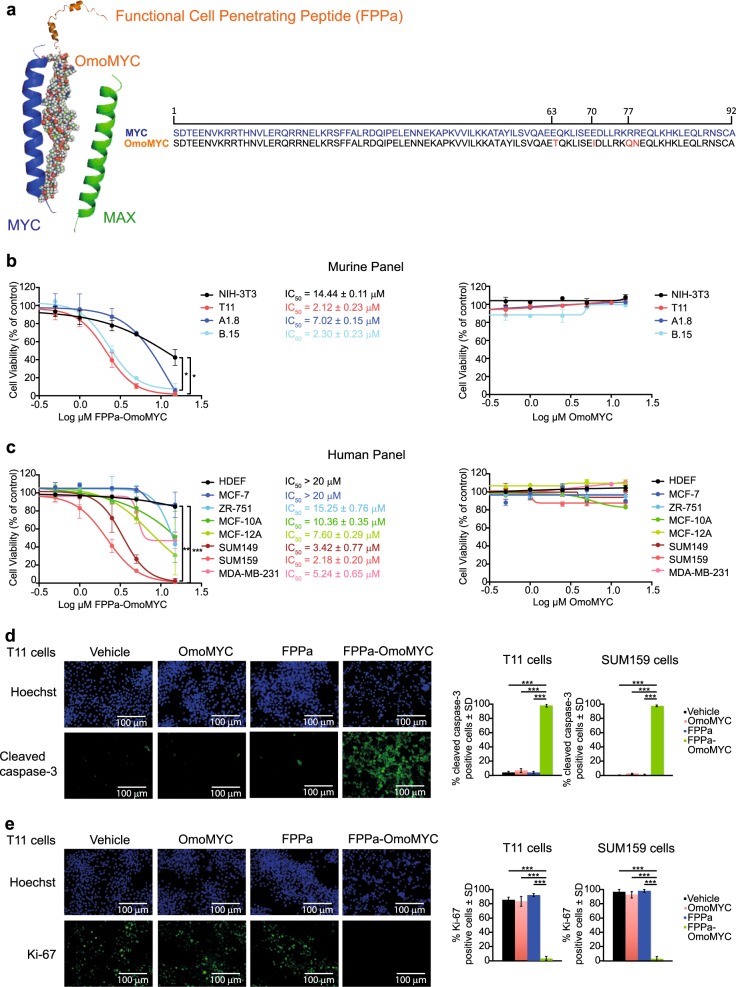
Treatment with FPPa-OmoMYC reduces cell viability, proliferation and induces apoptosis in TNBC cell lines. **a** Sequence and representative 3D structure of FPPa-OmoMYC interfering the interaction of MYC and MAX. Four amino acids substitutions that discriminate OmoMYC from MYC are shown in red. The FPPa sequence was selected from a Phylomer library promoting intracellular delivery as assessed by split-GFP complementation assay and protein production was as described [73]. The FPPa sequence is filed under the available patent numbers 2017902976 and 2017201163 **b** Murine and **c** Human cell line panel; cells were treated with increasing concentrations of FPPa-OmoMYC and OmoMYC for 24 h. T11, A1.8 and B.15 cell murine cell lines were kindly provided by C. Perou and L. Varticovski. NIH-3T3, HDEF, MCF-7, ZR-751, MDA-MB-231, MCF-10A, and MCF-12A were obtained from ATCC. SUM149, and SUM159 were purchased from Asterand Biosciences. All cell lines were tested for mycoplasma. Cells were seeded and treated for 24 h with increasing concentrations (0–15 µM) of FPPa-OmoMYC and OmoMYC. After treatments, cell viability was assessed using CellTiter-Glo® 2.0 (Promega). Luminescence signals were measured using the EnVision Multilabel Plate Reader (PerkinElmer Inc.; Waltham, MA, USA). IC_50_s were calculated and transformed 95% confidence intervals provided by GraphPad Prism 6 software analysis (GraphPad Software Inc., San Diego, CA, USA). IF assays showing cleaved caspase-3 (Cell Signalling Technology, #9661) (**d**) and proliferation (Ki-67, Cell Signaling Technology, #9449) (**e**) levels in T11 cells treated with FPPa-OmoMYC for 24 h at a concentration of 15 µM. Cells were seeded on coverslips. The following day, cells were treated with FPPa, OmoMYC, FPPa-OmoMYC, and vehicle (PBS) at a concentration of 15 µM for 24 h. Next, IF for Ki-67 (proliferation) and cleaved caspase-3 (apoptosis) was performed as previously described [32]. The IC_50_ values shown are mean ± SD from biological triplicate samples. All p-values were derived using two-tailed unpaired Student *t*-test where *, ** and *** represent *p* < 0.05, *p* < 0.005 and *p* < 0.0005, respectively relative to NIH-3T3 and HDEF

An alternative strategy to inhibit transcription factors takes advantage of peptide drugs, which, unlike small molecule inhibitors, have the potential to effectively block protein–protein interfaces that are relatively featureless. Specifically, interference peptides (iPeps) have been developed to specifically inhibit transcription factors requiring homodimerization and heterodimerization for transcriptional activity, such as homeodomain containing transcription factors overexpressed in TNBCs [31, 32]. Similarly, to specifically target MYC, a 92-amino acid bHLH-Zip protein designated as OmoMYC, was engineered as a dominant-negative MYC inhibitor. OmoMYC mimics the bHLH-Zip domain of MYC by incorporating four point mutations (E63T, E70I, R77Q, R78N) in the leucine zipper region (Fig. 1a) and thus prevents MYC heterodimerizing with MAX and inhibiting transcription activation of specific target genes [33–35]. Although OmoMYC exhibited some therapeutic potential for cancer treatment, most studies have deployed retroviral vectors or transgenic models which are not suitable for clinical translation [35–40]. OmoMYC on its own displays poor delivery across physiological barriers to the desired cellular compartment and thus, despite decades of active research, the therapeutic use of OmoMYC has been impaired by the lack of tumor cell penetration in vivo [30].

Herein, we describe the engineering of OmoMYC with an N-terminal functional penetrating Phylomer (FPP) (Hoffmann *et al.*, unpublished). The FPP sequence was derived from a structurally diverse Phylomer phage-display library comprising hundreds of millions of sequences (so-called Phylomers) of microbial and viral genomic origin [41–44]. The hallmark of this new generation of cell penetrating sequences is their capacity to evade late endosomal trapping and thus substantially enhancing both the intracellular delivery and the functionality of peptide drugs targeting intracellular ligands. In addition, the cell penetrating peptides derived from Phylomer libraries are not inherently associated with adverse immunoresponses. Shorter Phylomers such as those identified as cell penetrating peptides, have a lower stochastic likelihood of having MHC-binding T-cell epitopes [41]. And these particular peptides are made of L-amino acids which confer enhanced invisibility against proteases leading to a decreased immune system response mediated by antigen-presenting cells [45]. Here we demonstrated for the first time high efficacy of FPP-OmoMYC to inhibit TNBC growth with an IC_50_ concentration of ~1 µM, and thus at least one order of magnitude superior to known small molecule MYC inhibitors, with negligible effect in non-transformed cells. Importantly, FPP-OmoMYC exhibited potent anti-tumor effect in vivo in an aggressive TNBC allograft model, even after the cessation of treatment. Lastly, we discovered potent synergistic interactions between FPP-OmoMYC and docetaxel, doxorubicin and cetuximab which could be implemented for future treatment of MYC-activated cancers.

## Results and discussion

To assess the efficacy and selectivity of inhibiting MYC with interfering peptides in breast cancer cells, we engineered OmoMYC with an N-terminal FPP (namely FPPa, Fig. 1a). The resulting fusion, FPPa-OmoMYC, and controls (FPPa and OmoMYC in absence of the cell penetration sequence FPPa) were first tested in a panel of murine and human breast cancer cell lines as well as in normal cells.

We found that OmoMYC, in the absence of FPPa, had no significant effect on cell viability in any of the cell lines tested (Fig. 1b, c right). Similarly, the FPPa sequence on its own lacked biological activity (Supplementary Fig. 1a and b). In contrast, FPPa-OmoMYC effectively inhibited the growth of the highly aggressive stem cell marker-enriched claudin-low T11 (p53^−/^^−^) [43, 46] and basal-like A1.8 and B.15 (BRCA1^−^^/^^−^) [47] TNBC murine cell lines while having little effect on mouse embryonic fibroblasts (NIH-3T3) (Fig. 1b left). This suggested that FPPa was necessary for conferring anti-cancer activity to OmoMYC. While a few reports have explored peptide-based cell penetration agents [48] or biopolymers [49] as delivery vehicles for MYC-dominant negative peptides, >10 µM concentration and >10 days were required for biological activity. In contrast, we observed that FPPa-OmoMYC significantly reduced cell viability at 24 h (h) in the TNBC claudin-low MDA-MB-231 and SUM159 and basal-like SUM149 cell lines with low micromolar (1–2 µM) inhibitory dose fifty values (IC_50_s), while dermal epithelial fibroblasts (HDEF), normal-like epithelial cells (MCF-10A and MCF-12A) and less aggressive luminal-like cells (MCF-7 and ZR-751) significantly remained less affected by the treatment (Fig. 1c left). In contrast, all MYC inhibitors reported so far caused mainly cell growth arrest but not substantial cell death even when delivered at very high concentrations (10–65 µM) and for long treatment periods (>11 days) [24–26, 48, 49]. This suggested that FPPa-OmoMYC preferentially targets highly aggressive TNBC cell lines with unprecedented potencies and with negligible effect in non-transformed cells. This is consistent with reports demonstrating high sensitivity to MYC inhibition in TNBC overexpressing *MYC* [50]. The differential response between cell lines could be attributed to many factors, since MYC drives multiple physiological processes in different cell types. It has been shown that TNBCs possess higher MYC levels relative to that in other breast cancer subtypes. TNBC could therefore be more “addicted” to MYC oncogenic signaling for survival. Thus, this breast cancer subtype may be particularly sensitive to specific MYC inhibition. We further confirmed potent anti-cancer activity of FPPa-OmoMYC in T11 and SUM159 cells by a cleaved caspase-3 assay, which demonstrated very strong cell death induction, with ~97% of cells undergoing apoptosis after FPPa-OmoMYC treatment (Fig. 1d; Supplementary Fig. 2a), whereas only basal levels of apoptosis were observed with the control peptides FPPa and OmoMYC. Notably, T11 and SUM159 are p53-deficient TNBC cell lines. Our observation supports previous findings showing that OmoMYC enhanced MYC-induced apoptosis in myoblasts [34] and glioma cells [37] in a p53 independent fashion [51]. As expected, FPPa-OmoMYC, but not FPPa or OmoMYC alone, significantly inhibited 82% of cell proliferation (Ki-67) in T11 cells and 94% in SUM159 cells relative to that of vehicle-treated cells (Fig. 1e; Supplementary Fig. 2b).

**Fig. 2:**
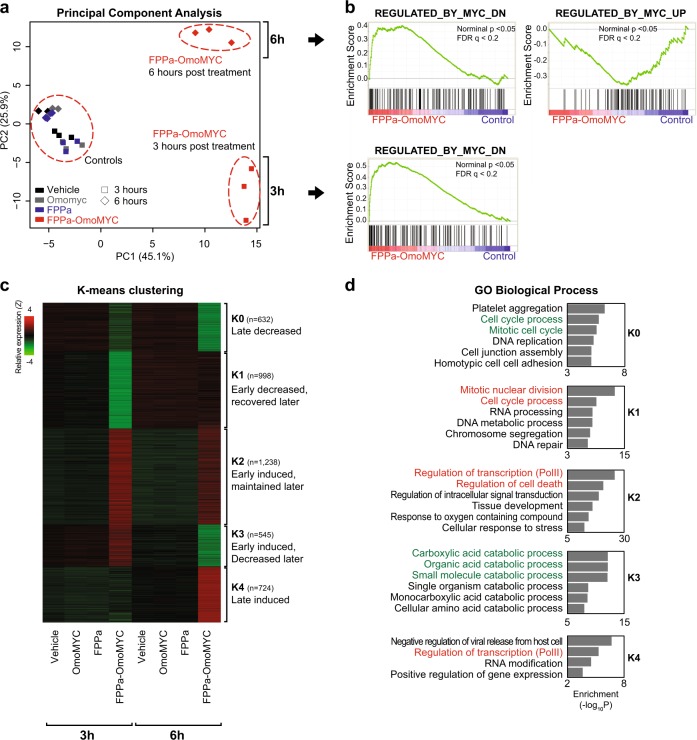
Extracellular delivery of FPPa-OmoMYC induces global gene expression changes. **a** PCA of global transcriptome from T11 cells treated with vehicle, FPPa, OmoMYC, and FPPa-OmoMYC, for 3 and 6 h. PCA was generated using R packages to assess variance expression patterns between controls and treatment groups. T11 cells were seeded, followed by 3 and 6 h treatment with vehicle (PBS), FPPa, OmoMYC, and FPPa-OmoMYC, at a concentration of 5 µM. Total RNA was then extracted from three biological replicates using RNeasy Mini Kit (Qiagen). All submitted samples at Australian Genome Research Facility (AGRF) in Perth had RNA integrity number of 10. Library preparation was carried out using Ilumina TruSeq mRNA Sample Preparation Kit according to manufacturer’s protocol at Phylogica Pty Ltd in Telethon Kids Institute. **b** GSEA of MYC target genes in FPPa-OmoMYC compared with vehicle at 6 h (upper panel) and 3 h (lower panel). GSEA was performed with the Signal2noise metric for ranking genes, 1000 permutations and the permutation type was set to Gene Set. The number of samples per phenotype was three. Sequencing of each library was performed on Illumina HiSeq using standard protocols at AGRF in Melbourne. The sequence reads were processed using Tuxedo tools and aligned to the mm9 mouse genome. **c** k-means clustering result showing at least two-fold differentially expressed genes, clustered into five groups (K0–K4, Bonferroni corrected FDR < 0.01). The colored bar on the left side of the heatmap shows overexpressed genes (red) and underexpressed (green) compared to counterparts. Differential gene expression between samples was quantified at the gene level using Cuffdiff in Cufflinks suite. The cut-off criteria were corrected using Bonferroni FDR 0.01 between any of the two sets out of nine experiments. The whole RNA sequencing study and data can be viewed at (GSE104553) and GSM2803244-67 **d** GO term analysis of top enriched biological pathways associated with K0 to K4 cluster genes. In green, cellular pathways associated with the differentially down-regulated genes and in red with the up-regulated genes

Next, we investigated the specificity of FPPa-OmoMYC in inhibiting MYC-dependent networks by RNA sequencing. TNBC T11 cells were treated with either FPPa-OmoMYC, FPPa, OmoMYC (at a 5 µM concentration) or vehicle (control) for 3 and 6 h, and processed by RNA extraction and sequencing. Principal component analysis (PCA) revealed that only FPPa-OmoMYC was able to induce significant changes in global gene expression relative to controls (Fig. 2a). We observed separate clusters for 3 and 6 h treatment groups, signifying sequential changes in gene expression profiles upon treatment. Gene set enrichment analysis (GSEA) revealed down-regulation of MYC activated sets [52] at 6 h post-treatment, while de-repression of MYC-repressed genes was observed at both 3 and 6 h time points (Fig. 2b). We identified five clusters of differentially expressed genes, designated as K0–K4. Gene Ontology (GO) analysis showed that cell cycle and cell division related processes were decreased during early and late stages (K0–K1), but a subset of these processes, including mitotic nuclear division, cell cycle and DNA metabolism recovered later (K1) (Fig. 2c, d). Various catabolic processes were induced at 3 h but decreased by 6 h (K3) and processes relating to RNA polymerase-II transcription were induced (K2 and K4). Notably, genes involved in the regulation of cell death were induced early and maintained at high levels at later time point (K2) (Fig. 2c). This supports the notion that more extended treatment may have even more profound effects. Recent studies have shown that the affinity of MYC-binding sites stratifies with different biological processes [53]. Low affinity sites are occupied only when MYC levels are high, as seen in various tumors [54], while high-affinity MYC sites are occupied by physiological levels of MYC and thought to be important for normal proliferating cells, perhaps explaining the low toxicity profile of the peptide for non-transformed cells. We also observed that the control peptides (FPPa, or OmoMYC alone) were unable to induce significant changes in the transcriptome. Our data provide molecular insights into the specificity of FPPa-OmoMYC in targeting cancer cells and its inhibition of MYC halts multiple facets of MYC function. This is in contrast with current inhibitors used in the clinic, such as anti-metabolites or CDK4 inhibitors [55, 56] which block only individual sets of MYC-dependent gene clusters. Collectively, these data support the notion that FPPa-OmoMYC regulates the tumor cell transcriptome in a MYC-dependent manner. In addition, we have outlined previously unknown dynamic changes of gene expression correlating with specific biological processes upon specific MYC inhibition via a peptide interference approach.

In order to validate that FPPa-OmoMYC competes with MYC for binding to MAX, we have performed immunoprecipitation experiments in T11 cells treated with the control conditions (OmoMYC and FPPa) and with FPPa-OmoMYC at 5 µM for 6 h (Supplementary Fig. 3). After the treatments, we pulled down endogenous MYC and immunoblotted the immunoprecipitated lysates against MAX. We observed that the treatment with FPPa-OmoMYC decreased the amount of bound MAX to MYC by 43.4% relative to that of the control condition OmoMYC. This confirmed that the active FPPa-OmoMYC peptide disrupted the interaction between MYC and its binding partner MAX and thus competes with MYC for binding to MAX.

**Fig. 3:**
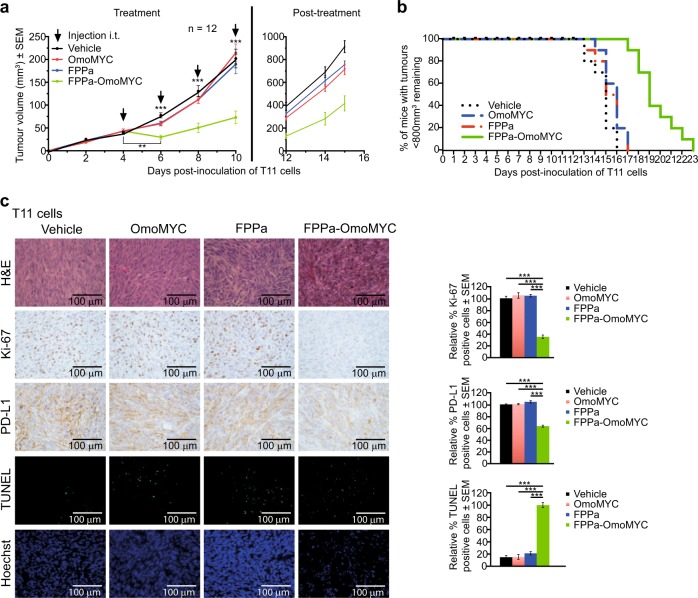
MYC inhibition with FPPa-OmoMYC confers therapeutic effect in a tumor allograft model in mice. **a** Left: Tumor volumes during the treatment phase of mice treated with vehicle, FPPa-OmoMYC, OmoMYC, and FPPa. Black arrows indicate injections. Right: Tumor volumes during the post-treatment phase. T11 cells stably expressing the luciferase gene were obtained using a retroviral expression vector. Retroviral particles were produced in HEK293T GAG-POL cells transfected with the retroviral packaging plasmids VSV-G. T11 cells were freshly infected with the supernatants a total of four times. All experimental animal work was performed in accordance with the Animal Ethics Committee of the University of Western Australia. Female BALB/cJ mice at 4 weeks of age were purchased from the Animal Resources Centre (WA, Australia). 2.5 × 10^5^ T11 cells were resuspended in 1:1 serum-free media: Matrigel (BD Bioscience, NSW, Australia) and injected subcutaneously into the flank. Once tumors reached 50 mm [3], 12 mice were randomly assigned for each group: vehicle (PBS), FPPa, OmoMYC, and FPPa-OmoMYC. 40 nmoles of peptides were intratumorally injected every two days for a total of four times. Tumors were measured by digital caliper and tumor volumes calculated with the formula: *V* = 0.5 × L × W^2^. Mice were sacrificed when the tumors were >800 mm^3^. **b** Percentage of mice with tumors < 800 mm^3^. **c** Images of H&E, Ki-67, PD-L1, and TUNEL stainings of representative allografts derived from vehicle, FPPa-OmoMYC, OmoMYC, and FPPa treated mice at day 12. H&E, Ki-67, PD-L1 (Abcam, ab174838), and TUNEL stainings in the tumors were performed as described [27]. Images are at 40× magnification. *, ** and *** represent *p* < 0.05, *p* < 0.005 and *p* < 0.0005 respectively, relative to FPPa-OmoMYC treated group

FPPa-OmoMYC acts by reducing the binding between MYC and MAX thus interfering with the transcriptional activity of MYC. Previously characterized MYC inhibitors are: H1, IIA6B17, 10058-F4, 10074-G5, and KJ-Pyr-9. These inhibitors disrupt either the binding of MYC with MAX (generally binding to MYC), or to the binding of MYC with the DNA. For instance, H1 binds to the H1 helix of the DNA binding domain of MYC. IIA6B17 possibly binds the helix-loop-helix (HLH) domain or the leucine zipper (HLH-ZIP) of either MYC or MAX but the exact binding site of the inhibitor has not been precisely determined. Similarly, 10058-F4 and 10074-G5 may bind in different regions of the HLH and HLH–ZIP domains of MYC. Finally, KJ-Pyr-9 was found to strongly bind to MYC, to the MYC-MAX heterodimer, and weakly to the MAX homodimer, but its binding site has not been precisely mapped. To investigate the efficacy of FPPa-OmoMYC in reducing breast cancer growth in vivo, we took advantage of a T11 allograft model, which faithfully models a highly proliferative and aggressive claudin-low TNBC [32, 46]. This model recapitulates highly aggressive claudin-low (mesenchymal) TNBC chemoresistant breast cancer. In contrast to other TNBC mice models such as human cell xenotransplants or humanized models, syngeneic T11 allografts carry an intact immune system. Consequently, we reasoned that this model has the advantage in that it mimics aggressive TNBC in patients. From day 1–4 post-inoculation of the cells, we observed a rapid increase of tumor burden to a volume of 50 mm^3^ (Fig. 3a). During treatment (day 4–10), a small but significant effect (*p* = 0.021) was observed when delivering OmoMYC (day 6). However, mice treated with FPPa-OmoMYC showed the strongest therapeutic effect (day 6: vehicle vs FPPa-OmoMYC, *p* = 1.24 × 10^−6^). Importantly, at day 6, tumors treated with FPPa-OmoMYC exhibited a marked and significant shrinkage, 31%, as compared to day 4 (*p* = 3.57 × 10^−3^). Notably, at day 15 post-inoculation (post-treatment phase), FPPa-OmoMYC maintained a significant therapeutic effect, with an average tumor volume two-fold lower than control groups (vehicle:896.6 mm^3^, *p* = 7.13 × 10^−4^; FPPa:755.7 mm^3^, *p* = 2.76 × 10^−3^; OmoMYC:738.7 mm^3^, *p* = 7.78 × 10^−3^ and FPPa-OmoMYC:436.8 mm^3^). This antitumoral effect of FPPa-OmoMYC, which was maintained after cessation of treatment, suggests that treatment for a longer period, with more than four injections, may result in a more sustained anti-tumor effect. While the majority of mice treated with vehicle control, FPPa or OmoMYC reached experimental (ethical) endpoint by day 17 post-inoculation of the cells, FPPa-OmoMYC animals retained tumor volumes < 800 mm^3^ until day 22 (Fig. 3b). Consistently, it has also been shown that inactivation of MYC in tumors using inducible viral systems was sufficient for sustained tumor regression, growth arrest, and differentiation in an in vivo osteogenic sarcoma mouse model [57].

In summary, FPPa-OmoMYC demonstrated potent anti-cancer activity by reducing the growth of highly proliferative claudin-low breast carcinoma cells in vivo. Small molecules inhibiting MYC, such as Mycro3 and KJ-Pyr-9, have demonstrated anti-cancer activity in vivo in a Kras-induced pancreatic cancer model [58] and in a TNBC xenograft [25]. However, a very high dose of inhibitor (100 mg/Kg) and 30 days treatment were required to achieve therapeutic benefit. Importantly, our study demonstrated significant reduction in tumor volume after just the initial injection at a dose of 32.2 mg/Kg. This corresponds to 291 µM of the peptide, outlining the highest potency of our MYC inhibitor compared to previously reported inhibitors used so far in pre-clinical studies in vivo [58].

The therapeutic effect of FPPa-OmoMYC was further confirmed by analyzing the harvested tumor tissues at day 12 post-inoculation by immunofluorescence (IF) and immunohistochemistry (IHC) (Fig. 3c).

Similarly, we observed a significant reduction in tumor cell proliferation (Ki-67) in the FPPa-OmoMYC treated tumors compared to that of all the other groups (vehicle vs FPPa-OmoMYC, *p* = 7.55 × 10^−8^). Induction of apoptosis by FPPa-OmoMYC in the tumor sections was confirmed by TUNEL assay with a six-fold increase in positive cells relative to vehicle (*p* = 9.85 × 10^−8^) (Fig. 3c LAST PANEL). These results demonstrate that FPPa-OmoMYC, but not OmoMYC or FPPa, is able to inhibit tumor proliferation and induce apoptosis when administered orthotopically in highly aggressive T11 allografts. We found that some of the anti-tumor effects of FPPa-OmoMYC were maintained even after the cessation of the treatment, confirming previous findings in osteogenic sarcoma mouse model [57].

Lastly, to assess the expression of direct MYC targets in the tumors, we quantified the protein levels of PD-L1 after FPPa-OmoMYC treatment by IHC. PD-L1 is an immune checkpoint protein, recently found to be downregulated following MYC inactivation [59]. Consistently, our data demonstrated a highly significant decrease in the expression of this direct MYC target in tumors treated with FPPa-OmoMYC relative to vehicle (*p* = 5.16 × 10^−7^) (Fig. 3c).

**Fig. 4:**
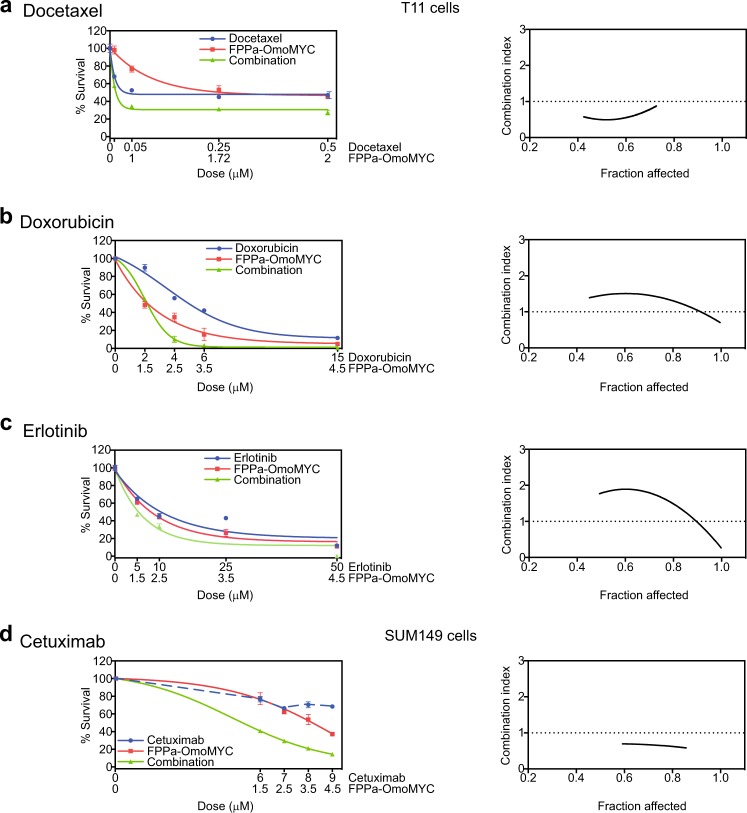
Evaluation of synergistic effects between FPPa-OmoMYC and chemotherapeutic drugs. Left: **a**–**c** Survival plots of T11 and **d** SUM149 cells treated with chemotherapeutic drugs alone or combined with FPPa-OmoMYC. Right: **a**–**d** Combination index plots. Chou and Talalay algorithm [74], included in the freely available CompuSyn software, was used to determine the nature of the interaction between the chemotherapeutic drug and FPPa-OmoMYC. Drugs were combined in constant ratios and the combination index (CI) value was determined. CI < 1 means synergism, CI = 1 means additivism and CI > 1 means antagonism. Values shown are mean ± SD from biological triplicate samples

MYC inhibitors have been investigated in combination with several chemotherapeutic drugs with the aim of reducing high drug dosage requirements for killing tumor cells, minimizing side effects while maintaining cytotoxic potential. For example, BET inhibitors were shown to sustain PI3K inhibition after lapatinib (EGFR inhibitor) treatment in breast cancer, enhancing their therapeutic potential [60, 61]. Additionally, MYC antisense oligonucleotides have been combined with cisplatin in melanoma [62], 10058-F4 and JQ-1 with the BCL2 inhibitor ABT-199 in lymphoma cells [63], and 10058-F4 with doxorubicin in leukemia cells [64]. To investigate whether FPPa-OmoMYC was able to sensitize TNBC cells to chemotherapy, four chemotherapeutic drugs commonly used in the clinic for the treatment of metastatic cancers, including breast [65–67] and head and neck carcinoma [68] were chosen (taxane: docetaxel; anthracycline: doxorubicin; epidermal growth factor receptor (EGFR): erlotinib; and monoclonal antibody: cetuximab). These inhibitors were tested alone or in combination with FPPa-OmoMYC (Fig. 4a–d). We found that T11 and SUM149 cells responded to the combinations of docetaxel + FPPa-OmoMYC and cetuximab + FPPa-OmoMYC, respectively, with a combination index (CI) lower than 1, indicating highly synergistic interactions (Fig. 4a and d right). In addition, doxorubicin and erlotinib showed synergistic CI values with FPPa-OmoMYC only at high drug concentrations (Fig. 4b and c right). These data indicated that FPPa-OmoMYC sensitized TNBC cells to the effects of chemotherapeutic agents, particularly for docetaxel and cetuximab. Clinical studies have indeed confirmed that docetaxel is highly efficacious when administered in MYC-activated TNBC patients [69]. Likewise, cetuximab could be used in conjunction with FPPa-OmoMYC to selectively target TNBC which frequently exhibit EGRF overexpression [70]. MYC has been shown to be associated with chemoresistance, particularly in TNBC, which has been extensively reported elsewhere in the literature [71]. The indirect MYC inhibitors, BET inhibitors, JQ1 and I-BET151, combined with lapatinib (an EGFR/ERBB2 inhibitor), demonstrated a cytotoxic synergistic effect in breast cancer in vitro and in vivo through the prevention of PI3K/AKT reactivation [60, 61]. Lastly the MYC antagonist polypeptide Penetratin-elastin like polypeptide-H1 was used in combination with doxorubicin in MCF-7 cells and resulted in a drug sensitization effect attributed to the decrease in the mRNA levels of polyamine synthesizing enzyme ornithine decarboxylase, which is at the same time a gene controlled by MYC [72]. These previous works are consistent with the synergism observed between FPPa-OmoMYC and cytotoxic chemoterapies.

In summary, we report for the first time, a potent delivery approach for OmoMYC with potential use in cancer therapy. Fusion of FPPa to OmoMYC led to unprecedented enhancement of MYC inhibition, tumor growth inhibition, and biological activity at the tumor site. Brief treatment of tumor cells with FPPa-OmoMYC led to a potent and specific activation of anti-proliferative and pro-apoptotic programs in highly aggressive TNBC cells both in vitro and in vivo. Our study has important implications for the design of a clinically relevant therapeutic approach for inhibiting MYC in highly aggressive and chemoresistant cancers, such as TNBC, for which no targeted therapy is available. Further re-engineering of FPPa-OmoMYC with targeting motifs could both enhance tumor cell specificity and/or provide target customization to specific subtypes of MYC-addicted cancers. Future investigations should expand this work with the design of targeted interference peptides engineered with tumor-specific homing sequences to enable tumor homing upon systemic administration.

## Electronic supplementary material

Supplementary Figure 1

Supplementary Figure 2

Supplementary Figure 3

Supplementary Information

## References

[1] Li Z, Van Calcar S, Qu C, Cavenee WK, Zhang MQ, Ren B (2003). A global transcriptional regulatory role for c-Myc in Burkitt’s lymphoma cells. Proc Natl Acad Sci USA.

[2] Mateyak MK, Obaya AJ, Sedivy JM (1999). c-Myc Regulates Cyclin D-Cdk4 and -Cdk6 Activity but Affects Cell Cycle Progression at Multiple Independent Points. Mol Cell Biol.

[3] Gebhardt A, Frye M, Herold S, Benitah SA, Braun K, Samans B (2006). Myc regulates keratinocyte adhesion and differentiation via complex formation with Miz1. J Cell Biol.

[4] Shen L, O’Shea JM, Kaadige MR, Cunha S, Wilde BR, Cohen AL (2015). Metabolic reprogramming in triple-negative breast cancer through Myc suppression of TXNIP. Proc Natl Acad Sci USA.

[5] Baudino TA, McKay C, Pendeville-Samain H, Nilsson JA, Maclean KH, White EL (2002). c-Myc is essential for vasculogenesis and angiogenesis during development and tumor progression. Genes Dev.

[6] Shi Y, Glynn JM, Guilbert LJ, Cotter TG, Bissonnette RP, Green DR (1992). Role for c-myc in activation-induced apoptotic cell death in T cell hybridomas. Science.

[7] Dang CV (2012). MYC on the path to cancer. Cell.

[8] Grushko TA, Dignam JJ, Das S, Blackwood AM, Perou CM, Ridderstrale KK (2004). MYC is amplified in BRCA1-associated breast cancers. Clin Cancer Res.

[9] Perou CM, Sørlie T, Eisen MB, van de Rijn M, Jeffrey SS, Rees CA (2000). Molecular portraits of human breast tumors. Nature.

[10] Wolfer A, Wittner BS, Irimia D, Flavin RJ, Lupien M, Gunawardane RN (2010). MYC regulation of a “poor-prognosis” metastatic cancer cell state. Proc Natl Acad Sci USA.

[11] Horiuchi D, Kusdra L, Huskey NE, Chandriani S, Lenburg ME, Gonzalez-Angulo AM (2012). MYC pathway activation in triple-negative breast cancer is synthetic lethal with CDK inhibition. J Exp Med.

[12] Green AR, Aleskandarany MA, Agarwal D, Elsheikh S, Nolan CC, Diez-Rodriguez M (2016). MYC functions are specific in biological subtypes of breast cancer and confers resistance to endocrine therapy in luminal tumours. Br J Cancer.

[13] Palaskas N, Larson SM, Schultz N, Komisopoulou E, Wong J, Rohle D (2011). 18F-fluorodeoxy-glucose positron emission tomography marks MYC-overexpressing human basal-like breast cancers. Cancer Res.

[14] Miller TW, Balko JM, Ghazoui Z, Dunbier A, Anderson H, Dowsett M (2011). A gene expression signature from human breast cancer cells with acquired hormone independence identifies MYC as a mediator of antiestrogen resistance. Clin Cancer Res.

[15] Dang CV, Reddy EP, Shokat KM, Soucek L (2017). Drugging the ‘undruggable’ cancer targets. Nat Rev Cancer.

[16] Bid HK, Phelps DA, Xaio L, Guttridge DC, Lin J, London C (2016). The bromodomain BET inhibitor JQ1 suppresses tumor angiogenesis in models of childhood sarcoma. Mol Cancer Ther.

[17] Delmore JE, Issa GC, Lemieux ME, Rahl PB, Shi J, Jacobs HM (2016). BET bromodomain inhibition as a therapeutic strategy to target c-Myc. Cell.

[18] Berthon C, Raffoux E, Thomas X, Vey N, Gomez-Roca C, Yee K (2016). Bromodomain inhibitor OTX015 in patients with acute leukaemia: a dose-escalation, phase 1 study. Lancet Haematol.

[19] Shu S, Lin CY, He HH, Witwicki RM, Tabassum DP, Roberts JM (2016). Response and resistance to BET bromodomain inhibitors in triple-negative breast cancer. Nature.

[20] Kurimchak AM, Shelton C, Duncan KE, Johnson KJ, Brown J, O’Brien S (2016). Resistance to BET bromodomain inhibitors is mediated by Kinome reprogramming in ovarian cancer. Cell Rep.

[21] Shi X, Mihaylova VT, Kuruvilla L, Chen F, Viviano S, Baldassarre M (2016). Loss of TRIM33 causes resistance to BET bromodomain inhibitors through MYC- and TGF-beta-dependent mechanisms. Proc Natl Acad Sci USA.

[22] Blackwood EM, Eisenman RN (1991). Max: a helix-loop-helix zipper protein that forms a sequence-specific DNA-binding complex with Myc. Science.

[23] Berg T, Cohen SB, Desharnais J, Sonderegger C, Maslyar DJ, Goldberg J (2002). Small-molecule antagonists of Myc/Max dimerization inhibit Myc-induced transformation of chicken embryo fibroblasts. Proc Natl Acad Sci USA.

[24] Yin X, Giap C, Lazo JS, Prochownik EV (2003). Low molecular weight inhibitors of Myc-Max interaction and function. Oncogene.

[25] Hart JR, Garner AL, Yu J, Ito Y, Sun M, Ueno L (2014). Inhibitor of MYC identified in a Krohnke pyridine library. Proc Natl Acad Sci USA.

[26] Clausen DM, Guo J, Parise RA, Beumer JH, Egorin MJ, Lazo JS (2010). In vitro cytotoxicity and in vivo efficacy, pharmacokinetics, and metabolism of 10074-G5, a novel small-molecule inhibitor of c-Myc/Max dimerization. J Pharmacol Exp Ther.

[27] Wang H, Hammoudeh DI, Follis AV, Reese BE, Lazo JS, Metallo SJ (2007). Improved low molecular weight Myc-Max inhibitors. Mol Cancer Ther.

[28] Fletcher S, Prochownik EV (2015). Small-molecule inhibitors of the Myc oncoprotein. Biochim Biophys Acta.

[29] Guo J, Parise RA, Joseph E, Egorin MJ, Lazo JS, Prochownik EV (2009). Efficacy, pharmacokinetics, tissue distribution, and metabolism of the Myc-Max disruptor, 10058-F4 [Z,E]-5-[4-ethylbenzylidine]-2-thioxothiazolidin-4-one, in mice. Cancer Chemother Pharmacol.

[30] Whitfield JR, Beaulieu ME, Soucek L (2017). Strategies to inhibit Myc and their clinical applicability. Front Cell Dev Biol.

[31] Beltran AS, Graves LM, Blancafort P (2014). Novel role of Engrailed 1 as a prosurvival transcription factor in basal-like breast cancer and engineering of interference peptides block its oncogenic function. Oncogene.

[32] Sorolla A, Ho D, Wang E, Evans CW, Ormonde CFG, Rashwan R (2016). Sensitizing basal-like breast cancer to chemotherapy using nanoparticles conjugated with interference peptide. Nanoscale.

[33] Soucek L, Helmer-Citterich M, Sacco A, Jucker R, Cesareni G, Nasi S (1998). Design and properties of a Myc derivative that efficiently homodimerizes. Oncogene.

[34] Soucek L, Jucker R, Panacchia L, Ricordy R, Tato F (2002). Nasi S. Omomyc, a potential Myc dominant negative, enhances Myc-induced apoptosis. Cancer Res.

[35] Savino M, Annibali D, Carucci N, Favuzzi E, Cole MD, Evan GI (2011). The action mechanism of the Myc inhibitor termed Omomyc may give clues on how to target Myc for cancer therapy. PLoS One.

[36] Soucek L, Whitfield JR, Sodir NM, Masso-Valles D, Serrano E, Karnezis AN (2013). Inhibition of Myc family proteins eradicates KRas-driven lung cancer in mice. Genes Dev.

[37] Annibali D, Whitfield JR, Favuzzi E, Jauset T, Serrano E, Redonno-Campos S (2014). Myc inhibition is effective against glioma and reveals a role for Myc in proficient mitosis. Nat Commun.

[38] Galardi S, Savino M, Scagnoli F, Pellegatta S, Pisati F, Zambelli F (2016). Resetting cancer stem cell regulatory nodes upon Myc inhibition. EMBO Report.

[39] Soucek L, Nasi S, Evan GI (2004). Omomyc expression in skin prevents Myc-induced papillomatosis. Cell Death Differ.

[40] Mongiardi MP, Savino M, Bartoli L, Beji S, Nanni S, Scagnoli F (2015). Myc and Omomyc functionally associate with the Protein Arginine Methyltransferase 5 (PRMT5) in glioblastoma cells. Sci Rep.

[41] Watt PM (2006). Screening for peptide drugs from the natural repertoire of biodiverse protein folds. Nat Biotechnol.

[42] Watt PM, Milech N, Stone SR (2017). Structure-diverse Phylomer libraries as a rich source of bioactive hits from phenotypic and target directed screens against intracellular proteins. Curr Opin Chem Biol.

[43] Prat A, Parker JS, Karginova O, Fan C, Livasy C, Herschkowitz JI (2010). Phenotypic and molecular characterization of the claudin-low intrinsic subtype of breast cancer. Breast Cancer Res.

[44] Milech N, Watt P (2012). The construction of “phylomer” peptide libraries as a rich source of potent inhibitors of protein/protein interactions. Methods Mol Biol.

[45] Watt PM (2009). Phenotypic screening of phylomer peptide libraries derived from genome fragments to identify and validate new targets and therapeutics. Future Med Chem.

[46] Roberts PJ, Usary JE, Darr DB, Dillon PM, Pfefferle AD, Whittle MC (2012). Combined PI3K/mTOR and MEK inhibition provides broad antitumor activity in faithful murine cancer models. Clin Cancer Res.

[47] Wright MH, Calcagno AM, Salcido CD, Carlson MD, Ambudkar SV, Varticovski L (2008). Brca1 breast tumors contain distinct CD44+/CD24- and CD133+cells with cancer stem cell characteristics. Breast Cancer Res.

[48] Giorello L, Clerico L, Pescarolo MP, Vikhanskaya F, Salmona M, Colella G (1998). Inhibition of cancer cell growth and c-Myc transcriptional activity by a c-Myc helix 1-type peptide fused to an internalization sequence. Cancer Res.

[49] Bidwell GL, Raucher D (2005). Application of thermally responsive polypeptides directed against c-Myc transcriptional function for cancer therapy. Mol Cancer Ther.

[50] Camarda R, Zhou AY, Kohnz RA, Balakrishnan S, Mahieu C, Anderton B (2016). Inhibition of fatty acid oxidation as a therapy for MYC-overexpressing triple-negative breast cancer. Nat Med.

[51] Hsu B, Marin MC, el-Naggar AK, Stephens LC, Brisbay S, McDonnell TJ (1995). Evidence that c-myc mediated apoptosis does not require wild-type p53 during lymphomagenesis. Oncogene.

[52] Zeller KI, Jegga AG, Aronow BJ, O’Donnell KA, Dang CV (2003). An integrated database of genes responsive to the Myc oncogenic transcription factor: identification of direct genomic targets. Genome Biol.

[53] Lorenzin F, Benary U, Baluapuri A, Walz S, Jung LA, von Eyss B (2016). Different promoter affinities account for specificity in MYC-dependent gene regulation. Elife.

[54] Jung LA, Gebhardt A, Koelmel W, Ade CP, Walz S, Kuper J (2017). OmoMYC blunts promoter invasion by oncogenic MYC to inhibit gene expression characteristic of MYC-dependent tumors. Oncogene.

[55] Liu T, Yu J, Deng M, Yin Y, Zhang H, Luo K (2017). CDK4/6-dependent activation of DUB3 regulates cancer metastasis through SNAIL1. Nat Commun.

[56] DeMichele A, Clark AS, Tan KS, Heitjan DF, Gramlich K, Gallagher M (2015). CDK 4/6 inhibitor palbociclib (PD0332991) in Rb+advanced breast cancer: phase II activity, safety, and predictive biomarker assessment. Clin Cancer Res.

[57] Jain M, Arvanitis C, Chu K, Dewey W, Leonhardt E, Trinh M (2002). Sustained loss of a neoplastic phenotype by brief inactivation of MYC. Science.

[58] Stellas D, Szabolcs M, Koul S, Li Z, Polyzos A, Anagnostopoulos C (2014). Therapeutic effects of an anti-Myc drug on mouse pancreatic cancer. J Natl Cancer Inst.

[59] Casey SC, Tong L, Li Y, Do R, Walz S, Fitzgerald KN (2016). MYC regulates the antitumor immune response through CD47 and PD-L1. Science.

[60] Stuhlmiller TJ, Miller SM, Zawistowski JS, Nakamura K, Beltran AS, Duncan JS (2015). Inhibition of lapatinib-induced Kinome reprogramming in ERBB2-positive breast cancer by targeting BET family bromodomains. Cell Rep.

[61] Stratikopoulos EE, Dendy M, Szabolcs M, Khaykin AJ, Lefebvre C, Zhou MM (2015). Kinase and BET inhibitors together clamp inhibition of PI3K signaling and overcome resistance to therapy. Cancer Cell.

[62] Leonetti C, Biroccio A, Candiloro A, Citro G, Fornari C, Mottolese M (1999). Increase of cisplatin sensitivity by c-myc antisense oligodeoxynucleotides in a human metastatic melanoma inherently resistant to cisplatin. Clin Cancer Res.

[63] Cinar M, Rosenfelt F, Rokhsar S, Lopategui J, Pillai R, Cervania M (2015). Concurrent inhibition of MYC and BCL2 is a potentially effective treatment strategy for double hit and triple hit B-cell lymphomas. Leuk Res.

[64] Pan XN, Chen JJ, Wang LX, Xiao RZ, Liu LL, Fang ZG (2014). Inhibition of c-Myc overcomes cytotoxic drug resistance in acute myeloid leukemia cells by promoting differentiation. PLoS ONE.

[65] Sharma P, Lopez-Tarruella S, Garcia-Saenz JA, Ward C, Connor CS, Gomez HL (2017). Efficacy of neoadjuvant carboplatin plus docetaxel in triple-negative breast cancer: combined analysis of two cohorts. Clin Cancer Res.

[66] Dickler MN, Rugo HS, Eberle CA, Brogi E, Caravelli JF, Panageas KS (2008). A phase II trial of erlotinib in combination with bevacizumab in patients with metastatic breast cancer. Clin Cancer Res.

[67] Henderson IC, Allegra JC, Woodcock T, Wolff S, Bryan S, Cartwright K (1989). Randomized clinical trial comparing mitoxantrone with doxorubicin in previously treated patients with metastatic breast cancer. J Clin Oncol.

[68] Bonner JA, Harari PM, Giralt J, Azarnia N, Shin DM, Cohen RB (2006). Radiotherapy plus cetuximab for squamous-cell carcinoma of the head and neck. N Engl J Med.

[69] Pereira CBL, Leal MF, Abdelhay E, Demachki S, Assumpcao PP, de Souza MC (2017). MYC amplification as a predictive factor of complete pathologic response to docetaxel-based neoadjuvant chemotherapy for breast cancer. Clin Breast Cancer.

[70] Nielsen TO, Hsu FD, Jensen K, Cheang M, Karaca G, Hu Z (2004). Immunohistochemical and clinical characterization of the basal-like subtype of invasive breast carcinoma. Clin Cancer Res.

[71] Lee KM, Giltnane JM, Balko JM, Schwarz LJ, Guerrero-Zotano AL, Hutchinson KE (2017). MYC and MCL1 cooperatively promote chemotherapy-resistant breast cancer stem cells via regulation of mitochondrial oxidative phosphorylation. Cell Metab.

[72] Bidwell GL, Raucher D (2006). Enhancing the antiproliferative effect of topoisomerase II inhibitors using a polypeptide inhibitor of c-Myc. Biochem Pharmacol.

[73] Milech N, Longville BA, Cunningham PT, Scobie MN, Bogdawa HM, Winslow S (2015). GFP-complementation assay to detect functional CPP and protein delivery into living cells. Sci Rep.

[74] Chou TC, Talalay P (1984). Quantitative analysis of dose-effect relationships: the combined effects of multiple drugs or enzyme inhibitors. Adv Enzym Regul.

