# Supplementary figures and images for: Tumor penetrating peptides inhibiting MYC as a potent targeted therapeutic strategy for triple-negative breast cancers
Source: Oncogene. 2018 Aug 3;38(1):140–50. doi: 10.1038/s41388-018-0421-y (PMC6318000; doi:10.1038/s41388-018-0421-y)

# Supplementary Figure 1

**a** T11 cells

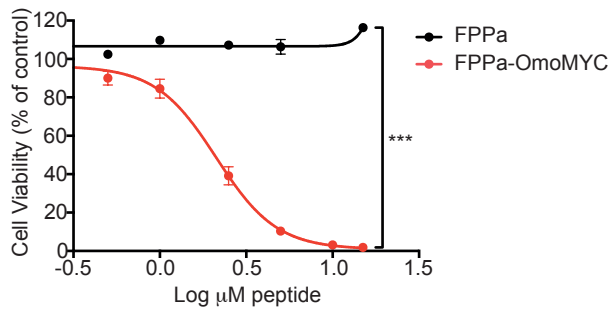

**b** SUM159 cells

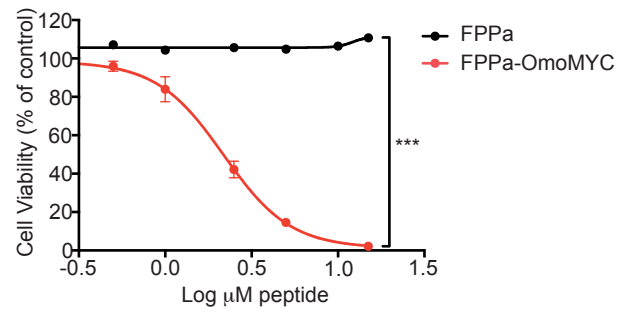

Supplement: Supplementary file 1 — Supplementary Figure 1 [file 41388_2018_421_MOESM1_ESM.pdf]

**a**

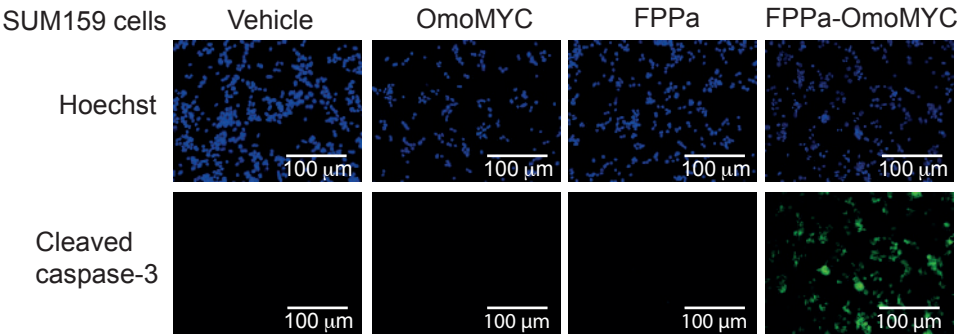

**b**

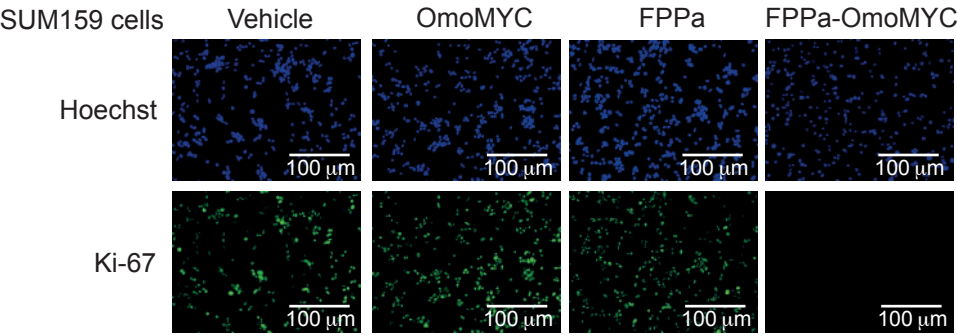

Supplement: Supplementary file 2 — Supplementary Figure 2 [file 41388_2018_421_MOESM2_ESM.pdf]
