# Supplementary material for: Tumor penetrating peptides inhibiting MYC as a potent targeted therapeutic strategy for triple-negative breast cancers
Source: Oncogene. 2018 Aug 3;38(1):140–50. doi: 10.1038/s41388-018-0421-y (PMC6318000; doi:10.1038/s41388-018-0421-y)

Supplementary Figure 3

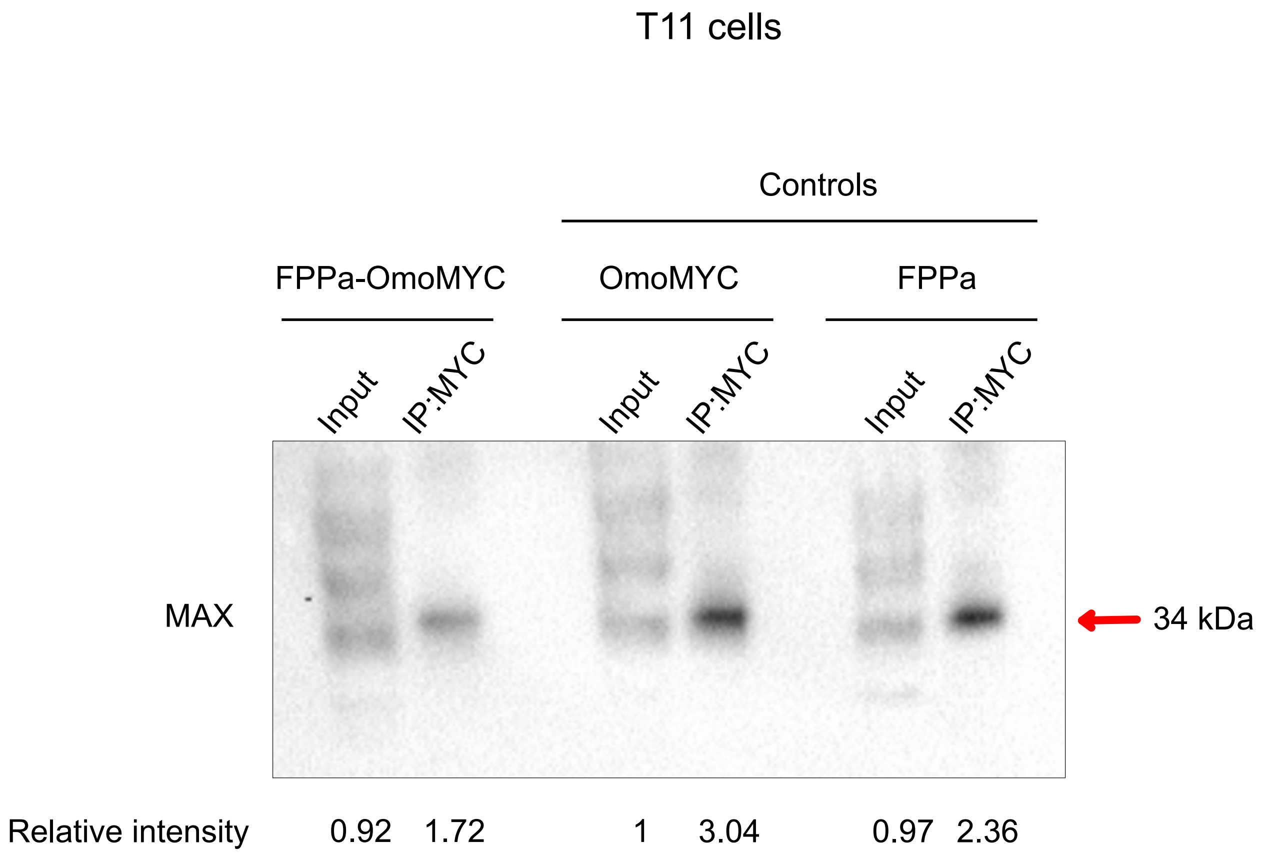

Supplement: Supplementary file 3 — Supplementary Figure 3 [file 41388_2018_421_MOESM3_ESM.pdf]
