# Supplementary material for: Tumor penetrating peptides inhibiting MYC as a potent targeted therapeutic strategy for triple-negative breast cancers
Source: Oncogene. 2018 Aug 3;38(1):140–50. doi: 10.1038/s41388-018-0421-y (PMC6318000; doi:10.1038/s41388-018-0421-y)
Supplement: Supplementary file 4 — Supplementary Information [file 41388_2018_421_MOESM4_ESM.pdf]

Supplementary Information

Figure Legend

Supplementary Figure 3. FPPa-OmoMYC competes with endogenous MYC for binding with MAX. T11 cells were treated with either control inactive peptides OmoMYC and FPPa or with the active FPPa-OmoMYC interference peptide, at a concentration of 5 μM for 6 hours. Cell lysates were immunoprecipitated with an anti-MYC antibody which does not recognize OmoMYC followed by immunoblotting with an anti-MAX antibody. The arrow indicates the 34 kDa anti-MAX product. The levels of MAX were calculated by determining the band intensities of MAX using the image Lab™ software. The relative value of MAX levels obtained are relative to the control input condition of OmoMYC which is assumed to be of value 1.

Cell culture and Treatment

T11 cells were grown to confluency in RPMI media supplemented with 10% FBS and 1% penicillin/streptomycin at 37°C and 5% CO_2. Cells were treated with 5µM of OmoMYC, FPPa and FPPa-OmoMYC for 6 hrs before being harvested using 0.25% trypsin for three minutes and centrifugation at 300xg for 3 minutes.

Lysis and Quantification

Pellets containing an equal number of cells were resuspended in 500 µl of lysis buffer containing 1 mM phenylmethylsulfonyl fluoride (cell signalling technology) and sonicated twice for 5 seconds each at amplitude 10 using a Misonix sonicator with a 419W microtip, resting on ice for 2 minutes between sonications. Samples were quantified as per manufacturers protocol (Biorad DC™ protein assay) and 600 µl of lysis buffer was added to 150 µl of lysate per immunoprecipitation, to adjust total protein to ~1 mg/ml.

Immunoprecipitation

4 µl of anti-MYC rabbit antibody (CST #9402S) was diluted in 200 µl of PBST (PBS + 0.1% Tween-20) and added to 50 µl of Dynabeads Protein G (ThermoFisher Scientific) and incubated with rotation at room temperature for 30 minutes. Beads were washed 3 times with 200 µl of PBST. 750 µl of each lysate was added to prepared beads and incubated for 6 hrs at 4°C with rotation. Myc-beads were added to FPPa-OmoMYC, OmoMYC and FPPa-treated lysates. Lysates were removed and the beads washed 3 times in PBS, resuspended in 100 µl of PBS and transferred to fresh tubes. Supernatant was removed and 20 µl of SDS-loading dye (10% glycerol, 0.02% bromophenol blue, 2% sodium dodecyl sulfate, 50 mM Tris-HCl pH 6.8) was added to each tube and heated at 70°C for 10 minutes. Beads were separated using a magnetic rack and analysed by SDS-PAGE.

Western Blot

Input samples were prepared by taking 15 µl of lysate used for the immunoprecipitations and 5 µl of 4x Laemmli buffer (Biorad) and heated at 70°C for 10 minutes. 10 µl of input and IP samples were loaded into a 4 – 15% gradient gel (Biorad precast mini-protean TGX gel). Proteins were transferred to a PVDF membrane using the transblot turbo system (Biorad) and blocked in 5% skim milk for 1 hr before being placed in anti-MAX mouse antibody (Abcam #53570) overnight at 4°C with gentle shaking. The membrane was washed 5 times in TBST for 5 minutes per wash at room temperature and incubated with Luminata Crescendo (Merck Millipore) for 5 minutes before imaging using the Chemidoc.
